# Biliary Surgery Via Minilaparotomy — A Limited Procedure for Biliary Lithiasis

**DOI:** 10.1155/1993/56128

**Published:** 1993

**Authors:** Takukazu Nagakawa

**Affiliations:** The Second Department of Surgery School of Medicine Kanazawa University Kanazawa Japan

## Abstract

Cholelithiasis until now has been treated using solvents, lithotripsy via a biliary endoscope, laser or shock wave lithotripsy, and laparoscopic cholecystectomy. have developed a new surgical treatment for cholelithiasis in which a cholecystectomy is performed through a minilaparotomy. This paper presents this new technique and discusses the principles of surgery for cholelithiasis using this technique. This procedure is performed by a 2 to 3 cm subcostal skin incision in the right hypochondrium. More than 400 patients were treated by this technique. This procedure is not different in terms of blood loss .or operation time from conventional methods, and no significant complications have occurred. Intraoperative X-ray examination is performed routinely because of easy insertion of a tube from the cystic duct into the bile duct. Reduction of the length of the incision greatly facilitates postoperative recovery, shortening the hospital length-of-stay to within 3 days. The surgical manipulation of only a limited area of the upper abdomen is unlikely to induce postoperative syndromes, such as adhesions or ileus. Following this experience, a biliary drainage procedure based on cholangionmanomery and primary closure of the choledochotomy was introduced. This approach allowed even patients with choledocholithiasis to undergo a minilaparotomy and be discharged within one week.


*HPB Surgery*, 1993, Vol. 6, pp. 255-262
Reprints available directly from the publisher
Photocopying permitted by license only

© 1993 Harwood Academic Publishers GmbH
Printed in the United States of America

### ACUTE FULMINANT PANCREATITIS: DEBRIDEMENT OR FORMAL RESECTION OF THE PANCREAS

HEIKKI KIVINIEMI, JYRKI MÄKELÄ and MATTI I. KAIRALUOMA
*Department of Surgery, Oulu University Central Hospital, Oulu, Finland*

*(Received 14 May 1992)*

During the ten year period from 1980 to 1989, 51 patients were treated at Oulu University Central Hospital for fulminant acute pancreatitis. Five were in a moribund state on admission and died shortly afterwards, 6 were treated conservatively and survived, and 40 were operated on, 17 by primary pancreatic resection and 23 by debridement of the peripancreatic area. Mortality rates were 53 per cent for the resection group and for the debridement group 22 per cent.

Reoperations were performed in 24 per cent of patients in the pancreatic resection group and in 60 per cent of those in the debridement group.

The high mortality rate associated with primary pancreatic resection has caused us to adopt a more conservative strategy, and surgical treatment is directed towards later complications of this severe disease.

KEY WORDS: Acute fulminant pancreatitis, pancreatic resection, debridement, reoperation

### INTRODUCTION

Despite the progress achieved in the medical care of patients with acute fulminant pancreatitis, the place of operation in relation to this disease remains controversial[1]. It is evident that the avoidance of operation in those with mild acute pancreatitis according to objective criteria has been one of the most important benefits to be obtained from clinical research into acute pancreatitis in recent decades.

There are many factors which have confused the situation regarding the role of operation in acute fulminant pancreatitis[2]. The clinical course is not always predictable, the lack of an accepted classification for the sequelae of acute pancreatitis makes comparisons between series difficult, and the timing and extent of the operation are also problematic.

Computed tomography scan (CT)[3] and C-reactive protein levels (CRP)[4] gained an established position in the diagnosis and follow-up of acute fulminant pancreatitis during the 1980s. Our purpose is to present our experience with this disease over the period 1980-1989, during which we used both debridement and pancreatic resection to treat it.

Address correspondence to: Heikki Kiviniemi, M.D., Department of Surgery, Oulu University Central Hospital, Kajaanintie 50, SF-90220 Oulu, Finland.

### PATIENTS AND METHODS

A total of 382 patients were treated for acute pancreatitis at Oulu University Central Hospital during the ten-year period from 1980 to 1989, 271 males and 111 females. Alcohol was an aetiological factor in 225 cases, gallbladder stones in 117 and other causes, mostly idiopathic, in 40. This group of patients included 51 (39 males and 12 females) who had a fulminant form of acute pancreatitis, the diagnosis of which was based on physical signs, laboratory findings and CT and was confirmed at laparotomy or autopsy. Alcohol was an aetiological factor in 35 cases, gallbladder stones in 15 and endoscopic retrograde cholangiography in one. Ranson's criteria were established using a modified scheme[1]. Forty of these 51 patients were operated on and 11 were treated conservatively. The operative strategy was decided at laparotomy. The pancreas was exposed through the omental bursa. If there was macroscopic necrosis in the pancreas and if CT showed hypodense areas, this increased the likelihood of pancreatic resection.

The operative technique was as follows. The operation was performed through a midline or transverse epigastric incision. In the case of debridement, Kocher's manoeuvre was performed. The pancreas was exposed through the lesser sac, and the peritoneum over the upper and lower part of the pancreas was opened. The peripancreatic sludge was removed gently from the retroperitoneal areas. The gallbladder was examined but was not routinely removed.

Pancreatic resection was performed by mobilization of the spleen and pancreatic tail. The splenic vein and artery were ligated, and the pancreas was resected at the level of the portal vein. The splenic bed was then drained through the bed of the 12th rib.

### RESULTS

Of the 11 patients treated conservatively, six survived using total parenteral nutrition. CT showed hypodense areas in the pancreas and the CRP levels rose over 200 mg/L, but returned to normal after two to three weeks. Five patients were in such a moribund state that an operation was not feasible, and all of them died. Forty patients were treated surgically. Worsening of the general condition or the suspicion of a local complication such as an abscess were the most common indications (Table 1). Seventeen patients (14 male, 3 female) were treated primarily by pancreatic resection, mostly within seven days of admission (Table 2). In six cases CT was performed preoperatively, showing signs of hypodensity in the pancreatic parenchyma. In only one case was CT performed twice before the operation showing a progression of pancreatic hypodensity.

Twenty-three patients (18 male, 5 female) were treated primarily by debridement of the peripancreatic area, also mostly within 7 days of admission (Table 2). CT was performed in 10 of these patients, 4 of whom had hypodense areas, leading to pancreatic resection in 3 instances. In one case CT was performed twice before the operation but did not show any changes in the parenchymal density of the pancreas.

Patient characteristics are presented in Table 3. The symptoms before admission did not differ between the groups, but the length of hospital stay and the delay in

**Table 1** Indications for operation.

| | |
|---|---|
| Unsuccessful conservative treatment | 21 |
| Intra-abdominal sepsis | 8 |
| Pancreatic abscess | 6 |
| Biliary pancreatitis | 3 |
| Diagnosis uncertain | 3 |

**Table 2** Time from admission to operation.

| *Group* | *No.* | *Time* $\leq 1$ *day* | $\leq 2$ *days* | $\leq 3$ *days* | $\geq 3$ *days* |
|---|---|---|---|---|---|
| Resection | 17 | 7 | 4 | 3 | 3 |
| Debridement | 23 | 8 | 1 | 7 | 3 |
| Total | 40 | 15 | 5 | 10 | 10 |

**Table 3** Patient characteristics

| | | |
|---|---|---|
| ♂/♀ | 14/3 | 17/6 |
| Age (years) | 40±13 (29–71) | 53±18 (30–80) |
| Hospital stay (days) | 25.8±19.3 (1–70) | 36±24 (9–90) |
| Symptoms before hospitalization (days) | 2.3±2.01.8±2.08 | |
| Delay in hospital before operation (days) | 2.6±2.6 (0.25–10) | 4.2±5.6 (0.25–20) |

hospital before operation were on average longer in the debridement group (Table 3).

The numbers of reoperations and intra-abdominal complications in the groups are shown in Tables 4 and 5. Reoperations were performed in 4 cases in the resection group (24.0%) and 14 in the debridement group (60.0%), mostly because of suspicion of a septic focus. An intra-abdominal abscess was found in one of 4 patients in the resection group. A haematoma was found in one patient and the operative finding was negative in two. One patient with an abscess survived but developed a colonic fistula which required 3 further operations. Two patients died of sepsis. Although the number of reoperations in the debridement group was high, only one patient died. Mostly there was a septic focus found at operation. One pancreaticocutaneous fistula and one colocutaneous fistula required further operations. Two reoperations were performed for colonic necrosis. Nine patients in the resection group ultimately died (53.0%), mostly of multi-organ failure (seven patients), but with haemorrhage as the cause of death in two cases. Five patients in

**Table 4** Reoperations in the resection and debridement groups.

| *No. of reoperations* | *No. of patients* | *No.- of deaths* | *No. of patients* | *No. of deaths* |
|---|---|---|---|---|
| One | 3 | 2 | 7 | 1 |
| Two | 0 | — | 2 | — |
| Three | 1 | — | 3 | — |
| Four or more | 0 | — | 1 | — |
| No operation | 13 | 7 | 10 | 4 |
| Total | 17 | 9 (53%) | 23 | 5 (22%) |

**Table 5** Intra-abdominal complications

| *Complication* | *Group* *Debridement* | *Resection* |
|---|---|---|
| Intra-abdominal abscess | 5 | 1 |
| Intestinal fistula | 3 | 1 |
| Prolonged fever | — | 2 |
| Peripancreatic phlegmon | 1 | 1 |
| Wound infection | 1 | 1 |
| Pancreatic fistula | 1 | — |
| Necrosis of colostomy | 1 | — |
| Gastric retention | 1 | — |
| Total | 13 | 5 |

**Table 6** Ranson's criteria outcome

| | *Procedure* | | | |
|---|---|---|---|---|
| *No. of positive Ranson's signs* | *Resection No. of pts.* | *No. of deaths* | *Debridement No. of pts.* | *No. of deaths* |
| 0–2 | 5 | — | 10 | 1 |
| 3–4 | 6 | 3 | 10 | 2 |
| 5–6 | 6 | 6 | 3 | 2 |

the debridement group died, four without a reoperation. All of these were elderly females with severe gallstone disease. As mentioned earlier, one patient died after a reoperation (pancreatic resection). More patients in the pancreatic resection group had a serious form of the disease according to Ranson's criteria, and the patients with fewer positive signs recovered better (Table 6).

### DISCUSSION

The resection rate for all patients with acute pancreatitis varies from 4.6 to 10%,[5,6,7] so that our figure of 4.5% is in accordance with the general trend. If pancreatic resection after primary debridement is taken into account, however, our resection rate increases to 7%. In some reports patients undergoing pancreatic resection are considered together with those undergoing retroperitoneal debridement and drainage[8], but it would seem to us that a distinction should be made between debridement and formal pancreatic resection. The discrepancy may in part be caused by the amount of peripancreatic necrosis, which increases with time and may be difficult to separate from the pancreatic parenchyma itself.

The high mortality rate of about 50% among our patients with primary resection is in part explained by the critical condition of the patients and technical difficulties encountered during pancreatic resection. This explanation is supported by the high rate of multiple organ failure as a cause of death, this being the usual end point of this serious disease. Mortality rates from 22 to 33% for pancreatic resection have been reported earlier in cases of fulminant acute pancreatitis[6,7], and it has even been suggested that pancreatic resection is better than debridement[6]. In our opinion, this problem has not yet been resolved, as the numbers of patients even in recent reports, are too small to allow statistical comparisons to be made[6]. It has been suggested that pancreatic resection could prevent the toxic effects of pancreatic enzymes[9]. Early pancreatic resection does not seem to prevent multiple organ failure, however, either in our results or according to earlier findings[10].

The role of debridement is controversial. In the presence of pancreatic necrosis, where urgent pancreatic sequestrectomy has been suggested as being safer than formal pancreatic resection[11,12], others have suggested that the indications for pancreatic debridement are limited[13]. Our results show however that careful debridement and pancreatic sequestrectomy are sensible treatment alternatives.

The fact that reoperations were more common in the debridement group means that debridement alone cannot always stop the course of the disease. The good results achieved in this group reflected the high incidence of septic foci found at operation. Also, the disease was milder in the debridement group in terms of Ranson's signs. It has been shown that reoperations in patients with multiple organ failure without a local focus carry a bad prognosis[14].

Before CT scan was available for the diagnosis and evaluation of haemorrhagic pancreatitis, the operative tactics were determined by the macroscopic findings, which are unreliable for the evaluation of pancreatic necrosis[15]. When the advent of CT suggested that the maintenance of pancreatic blood flow was important, an increased interest was shown in removing that portion of the pancreas that had a poor circulation. Thus CT increased the frequency of pancreatic resection in our unit. The poor results we obtained with the present cases have now forced us to change our strategy towards a more conservative approach, which appears to be a common tendency in recent reviews[16]. Repeat CT scan was used preoperatively in two of our cases, one of which showed hypodensity in the pancreas. The fact that a deterioration of CT signs in serial scans has very seldom been observed[17,2] suggests that mild and severe pancreatitis may be different pathological entities from the outset.

Serial CRP determination during conservative treatment and after operation can help to predict the amount of necrosis in the pancreatic area. A CRP assay is not specific to acute pancreatitis and should always be used in conjunction with clinical criteria. The clinical state of the patient will also be the most important factor in the future when deciding whether or not to operate, but optimal surgical treatment is crucial.

### *References*

1. Ranson, J.H.C., Rifkind, K.M., Roses, D.F., Fink, S.B., Eng, K. and Spencer, F.C. (1974) Prognostic signs in the role of operative management in acute pancreatitis. *Surg. Gynecol. Obstet.*, **139**, 69
2. Lange, J.F., Teng, H.T., Menu, M. and Ham, A.G. (1988) The role of computed tomography in the management of acute pancreatitis. *Acta Chir. Scand.*, **154**, 461–465
3. Kivisaari, L., Somer, K., Standertskjold-Nordenstam, C.-G., Schröder, T., Kivilaakso, E. and Lempinen, M. (1983) Early detection of acute fulminant pancreatitis by contrast-enhanced computed tomography. *Scand. J. Gastroenterol.*, **18**, 39–41
4. Mayer, A.D., McMahon, M.J., Bowen, M. and Cooper, E.H. (1984) C-reactive protein: an aid to assessment and monitoring of acute pancreatitis. *J. Clin. Pathol.*, **37**, 207–211
5. Autio, V., Juusela, E., Lauslahti, K., Markkula, H. and Pessi, T. (1979) Resection of the pancreatitis for acute hemorrhagic and neurotizing pancreatitis. *World J. Surg.*, **3**, 631–639
6. Kivilaakso, E., Lempinen, M., Mäkeläinen, A., Nikki, P. and Schröder, T. (1984) Pancreatic resection versus peritoneal lavation for acute fulminant pancreatitis. *Ann. Surg.*, **199**, 426–431
7. Aldridge, M.C., Ornstein, M., Glazer, G. and Dudley, A.F. (1985) Pancreatic resection for severe acute pancreatitis. *Br. J. Surg.*, **72**, 796–800
8. White, T.T. and Heimbach, D.M. (1976) Sequestrectomy and hyperalimentation in the treatment of haemorragic pancreatitis. *Am. J. Surg.*, **132**, 270–275
9. Watts, G.T. (1963) Total pancreatectomy for fulminant pancreatitis. *Lancet*, **ii**, 384
10. Teerenhovi, O., Nordback, I. and Isolauri, J. (1988) Influence of pancreatic resection on systemic complications in acute necrotizing pancreatitis. *Br. J. Surg.*, **75**, 793–795
11. Wilson, C., McArdle, S.S., Carter, D.C. and Imrie, C.W. (1988) Surgical treatment of acute necrotizing pancreatitis. *Br. J. Surg.*, **75**, 1119–1123
12. Teerenhovi, O., Nordback, I. and Eskola, J. (1989) High volume lesser sac lavage in acute necrotizing pancreatitis. *Br. J. Surg.*, **76**, 370–373
13. Smadja, C. and Bismuth, M. (1988) Pancreatic debridement in acute necrotizing pancreatitis: an obsolete procedure. *Br. J. Surg.*, **73**, 408–410
14. Bunt, T.J. (1985) Urgent relaparothomy: The high-risk nor choice operation. *Surgery*, **98**, 555–559
15. Nordback, I., Pessi, T., Auvinen, O., *et al.* (1985) Determination of necrosis in necrotizing pancreatitis. *Br. J. Surg.*, **72**, 225–227
16. Poston, G.J. and Williamson, R.C.N. (1990) Surgical management of acute pancreatitis. *Br. J. Surg.*, **77**, 5–12
17. Balthazar, E.J., Ranson, J.H.C., Naidich, D.P., Megibow, A.J., Caccavale, R. and Cooper, M.M. (1985) Acute pancreatitis: Prognostic value of CT. *Radiology*, **156**, 767–772

*(Accepted by S. Bengmark 17 May 1992)*

### INVITED COMMENTARY

Dr Kiviniemi and his colleagues are to be commended for accumulating data on a large number of complex and difficult patients. They accurately identify the two principal problems in therapeutic operations for fulminant acute pancreatitis: (1) how can the clinical course be predicted in an individual patient and (2) what type of surgical intervention should be applied (and when) to alter the course in those whose condition is deteriorating? The authors further emphasize the difficulty in categorizing patients in a fashion that will allow comparison between different series.

This non-randomized retrospective review will not end the extensive debate in the literature on the above questions. Some of the techniques currently used in the treatment of fulminant acute pancreatitis were not widely employed in this series.

Thus CT scans were performed in only 16 of the 40 operated patients. The definition of fulminant acute pancreatitis is given in broad terms only, and while we know the Ranson's scores for the operated patients, these data are unavailable for the unoperated group and those deemed to have less severe pancreatitis. It is unclear exactly how the patients were allocated to the two treatment arms. Macroscopic necrosis and hypodense areas on CT scan "increased the likelihood of pancreatic resection", although either treatment might have been applicable in such situations. As the authors state, the debridement group contained more people with a low Ranson's score (20/23 with 0–4 signs) than the resection group (11/17) with 0–4 signs). Six of the nine patients in the resection group who died had 5–6 Ranson's signs, as opposed to two of the five patients in the debridement group who died. Morever those with resection underwent operation a mean of 1.6 days sooner; they either presented with more serious illness (as supported by their Ranson scores) or deteriorated more rapidly. Although the groups are not strictly comparable therefore, the lower mortality and reoperative rate in the debridement group will lend weight to the arguments of those who feel that pancreatic resection in fulminant acute pancreatitis carries too high a risk with too little benefit.

This same bias against early formal resection emerges from most but not all other series. A second group in Finland adopted a policy of pancreatic resection for necrotizing acute pancreatitis in 1973 and found a 45% mortality in 84 resections[1]. Pancreatectomy did not affect the early systemic complications of acute pancreatitis, and it was associated with a high incidence of late complications including diabetes in 92%[1,2]. By contrast a third group of Finnish surgeons have reported a randomized trial favouring resection over "peritoneal lavation", (i.e. debridement with lavage) in acute fulminant pancreatitis[3]. There were only four deaths among eighteen patients treated with resection (22%), whose mean Ranson's score was 3.7 (range 2–6). In one British series four of seven patients receiving subtotal pancreatectomy died and in another one an identical mortality rate for resection (four of seven) was bettered by a lower mortality rate (four of fourteen) for necrosectomy[4,5]. Apart from its inherent risk, another drawback of resection is the surgeon's difficulty in distinguishing pancreatic from peripancreatic necrosis[6,7]. Debridement avoids the problem by confining the "resection" to tissue that is dead, whatever its origin. Its use is supported by the low death rates in patients with severe acute pancreatitis both in Britain (21% mortality) and in Germany (8% mortality)[8,9].

In Dr Kiviniemi's experience, the incidence of complications (13/23, 56%), reoperations (13/23, 56%), and death (5/23, 23%) for patients undergoing debridement are in line with other similar series but remain much too high for surgeons to be content with this or any other current treatment plan for these patients. Further research into the basic pathophysiology of acute pancreatitis is required to develop agents that will prevent, interrupt or at least modulate the mechanisms of injury and alleviate the need for such drastic surgical measures.

R. Howerton
R.C.N. Williamson
HPB Unit, Department of Surgery
Hammersmith Hospital
Du Cane Road
London W12 0HS UK

### *References*

1. Teerenhovi, O., Nordback, I. and Isolauri, J. (1988) Influence of pancreatic resection on systemic complications in acute necrotizing pancreatitis. *Br. J. Surg.*, **75**, 793–795
2. Nordback, I.H. and Auvinen, O.A. (1985) Long term results after pancreas resection for acute necrotizing pancreatitis. *Br. J. Surg.*, **72**, 687–689
3. Kivilaakso, E., Lempinen, M., Makelainen, A., Nikki, P. and Schroder, T. (1984) Pancreatic Resection versus Peritoneal Lavation for Fulminant Acute Pancreatitis A Randomized Prospective Study. *Ann. Surg.*, **199**, 426–431
4. Aldridge, M.C., Ornstein, M., Glazier, G. *et al.* (1985) Pancreatic resection for severe acute pancreatitis. *Br. J. Surg.*, **72**, 796–800
5. Wilson, C., McArdle, C.S., Carter, D.C. *et al.* (1988) Surgical treatment of acute necrotizing pancreatitis. *Br. J. Surg.*, **75**, 1119–1123
6. Nordback, I., Pessi, T., Auvinen, O. *et al.* (1985) Determination of necrosis in necrotizing pancreatitis. *Br. J. Surg.*, **75**, 225–227
7. Nordback, I. and Larslahti, K. (1986) Clinical Pathology of Acute Necrotic Pancreatitis. *J. Clin. Pathol.*, **39**, 68–74
8. Larvin, M., Chalmers, A.G., Robinson, P.J. *et al.* (1989) Debridement and closed cavity irrigation for treatment of pancreatic necrosis. *Br. J. Surg.*, **76**, 465–471
9. Beger, H.G., Buchler, M., Bittner, R. *et al.* (1988) Necrosectomy and postoperative local lavage in patients with necrotizing pancreatitis: results of a prospective clinical trial. *World J. Surg.*, **12**, 255–262

